# DNA Mechanical Strain Steers Transcription Factor Recognition

**DOI:** 10.21203/rs.3.rs-7027346/v1

**Published:** 2026-05-12

**Authors:** Ariel Afek, Minyi Yao, Michael O’Hagan, Karn Onoon, Lihee Givon, Shelly Rogotner, Raul Salinas, Raj Nithun, Naama Kessler, Muhammad Jbara, Orly Dym, Tanadet Pipatpolkai, Maria Schumacher

**Affiliations:** Weizmann Institute of Science; Weizmann Institute of Science; Weizmann Institute of Science; Mahidol University; Weizmann Institute of Science; Weizmann Institute of Science; Duke University School of Medicine; Tel Aviv University; Weizmann Institute of Science; Tel Aviv University; Weizmann Institute of Science; Nanyang Technological University; Duke University

## Abstract

DNA is not merely a linear code of bases, but a mechanically constrained polymer whose backbone continuity restricts the conformations accessible during protein recognition. Yet, although base-sequence preferences have been extensively mapped across human transcription factor (TF) families, we lack comparable maps of how TFs read backbone continuity and DNA mechanics, leaving these layers of recognition poorly understood. Here we introduce PIC-NIC, a high-throughput platform that uses site-specific backbone nicks to perturb DNA mechanics while preserving base identity and minimizing accompanying chemical changes. Across 15 TFs spanning eight structural families, PIC-NIC reveals a highly position-dependent response to backbone disruption: most positions are permissive, whereas nicking at mechanically sensitive sites can substantially reshape binding. Mechanistic analyses integrating PIC-NIC maps with newly determined TF–DNA structures, structural comparisons, binding kinetics, and molecular simulations show that sensitive positions often coincide with strain-adapted DNA geometries, including Hoogsteen conformations, whose relaxation can enhance binding or rewire sequence specificity. Genomic single-strand breaks and repair maps further suggest that TF retention at nicked DNA may influence local repair-factor accessibility. Together, these findings systematically map DNA backbone mechanics as a position-resolved layer of TF recognition across diverse protein families, showing how this layer can reshape binding specificity and may influence repair-factor accessibility.

DNA is commonly represented as a linear string of letters corresponding to its base sequence, with transcription factor (TF) binding sites typically depicted as short sequence motifs derived from this onedimensional alphabet. Yet TFs bind a physical polymer, not an abstract code, encountering DNA as a three-dimensional structure whose bases, sugars, and phosphates are arranged along a continuous backbone with defined geometries and mechanical constraints^2,4–7^. Beyond establishing the double helical structure, this backbone also governs how chemical groups are positioned in space and which conformations DNA can adopt and sustain during protein recognition. Mechanics therefore becomes an integral part of the recognition mechanism.

This mechanical view is supported by structural and theoretical studies showing that DNA mechanics shapes protein–DNA interactions, from nucleosome wrapping to topoisomerase engagement^3,8–12^, and that protein–DNA complexes span a continuum from highly deformed, strain-adapted conformations^8,13,14^ to near-relaxed geometries^15–17^ (Fig. 1a).

This physical view raises a direct experimental question: which features of the DNA backbone and local duplex architecture contribute to protein recognition beyond base identity? Pioneering biochemical studies began to address this question by chemically perturbing different components of the DNA interface. Phosphate ethylation interference and missing-phosphate approaches mapped positions where phosphate groups contribute to binding, while missing-nucleoside and abasic-site strategies extended this logic to sugars, bases, and local duplex integrity^14,18–22^. Related substitutions, including methylphosphonates and phosphorothioates, further probed the role of backbone charge and phosphate chemistry^23^. Together, these approaches identified backbone-related contacts important for binding in selected protein–DNA complexes. In some TFs, including AP-2α, ETS1, and NF-κB, defined backbone perturbation experiments further showed that local disruption of backbone integrity can modulate TF binding, including cases where perturbation enhances binding^14,24^. Similar principles have been established in other DNA-binding protein systems, including restriction enzymes and DNA-modifying enzymes, where backbone geometry and phosphate contacts have been shown to play essential roles in sequence-specific recognition^25,26^. These studies provided an essential foundation for understanding recognition beyond direct base readout.

Despite their importance, these approaches left a critical gap in both scope and interpretability. Most were applied to selected proteins and binding sites, and therefore did not provide a broad, position-resolved view of backbone contributions comparable to what is now available for base-pair preferences (Extended Data Table 1a). Moreover, these perturbations often alter several DNA properties at once. Removing or modifying a phosphate can change charge, hydrogen-bonding capacity, steric structure, and local backbone geometry, whereas missing-nucleoside or abasic-site approaches can also perturb base contacts, stacking interactions, and duplex stability (Fig. 1b). Thus, although these experiments identify positions where backbone-associated features influence binding, they often cannot determine why: whether the effect reflects loss of phosphate chemistry, disruption of backbone continuity, altered local geometry, or relaxation of a strained DNA conformation^21^. As a result, for most eukaryotic TFs—including factors central to cell fate, oncogenesis, and transcriptional activation—the mechanical and chemical contributions of the DNA backbone remain difficult to dissect and far less characterized than sequencebased binding preferences.

To address this gap, we sought a more controlled strategy that perturbs backbone continuity while minimizing accompanying changes to DNA chemical identity (Extended Data Table 1b). Preserving the phosphate at the break site provides a way to examine the contribution of backbone continuity and local geometry while retaining key features of the native backbone (Fig. 1c, e). In parallel, comparison with phosphate-removing perturbations can help identify positions where phosphate chemistry itself contributes to binding. This logic forms the basis of PIC-NIC (Protein–DNA Interaction Characterization via Nick-Induced Conformations), a high-throughput platform designed to map how backbone continuity, phosphate chemistry, and DNA mechanical strain contribute to TF recognition in a position-resolved manner across multiple TFs.

## PIC-NIC Maps DNA Nick Sensitivity with Base and Phosphate Chemistry Preserved

**Figure 1:**
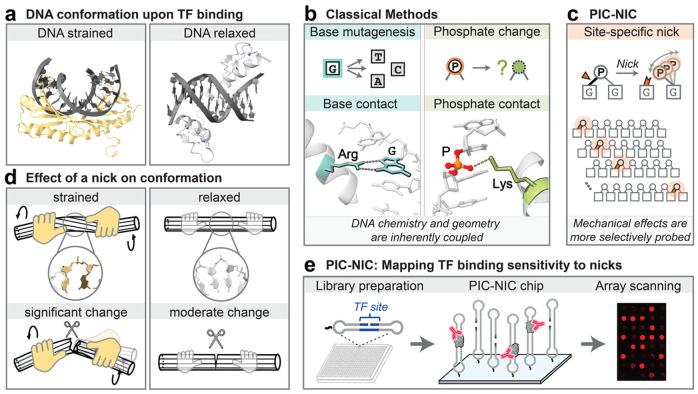
PIC-NIC enables high-throughput dissection of structural contributions to TF–DNA recognition. **(a)** TF binding can place DNA in distinct mechanical states. Some complexes substantially deform the duplex and store strain in the backbone (*left*; PDB ID: 1QNE), whereas some complexes bind DNA sites that remain close to the relaxed B-form geometry (*right*; PDB ID: 1AAY). **(b)** Traditional base mutagenesis (*left*) alters nucleotide identity to probe base-specific contributions, whilst phosphate removal (*right*), alters the chemical moiety to probe the importance of individual backbone phosphate. **(c)** PIC-NIC systematically extends this framework to site-specific single-strand breaks that preserve base identity and, in one design, retain phosphate composition while disrupting backbone continuity. This enables a more controlled assessment of how backbone continuity and local DNA geometry contribute to TF–DNA recognition beyond direct base readout. **(d)** A site-specific nick has different effects on different mechanical states. When the DNA is strain-loaded (*left*), cutting one strand disrupts the backbone continuity and allows the strained conformation to relax. When the bound DNA is already relaxed (*right*), the same nick releases little strain, and the conformation is largely preserved. **(e)** Overview of the PICNIC platform. Custom-designed nicked DNA libraries are immobilized on microarrays, incubated with target TFs and fluorescently labeled antibodies, and quantitatively scanned to assess binding at each nicked position.

The concept of using PIC-NIC to interrogate DNA mechanics is based on a simple mechanical principle (Fig. 1d): a break has the greatest effect when it releases strain stored in a constrained structure, whereas the same break has a smaller effect in an unstrained structure. By analogy, a nick in DNA is expected to have the strongest effects at positions where TF recognition depends on backbone continuity to establish or maintain a strained DNA geometry, while having more limited effects at positions where the bound DNA is mechanically relaxed.

Building on this principle, we generated PIC-NIC libraries containing defined single-strand breaks at each position across canonical TF-binding motifs and measured their effects on TF binding (Fig. 1c, e). In the primary PIC-NIC design, each nick retains the 5′ phosphate, preserving base identity and phosphate composition while selectively disrupting backbone continuity (Extended Data Fig. 1). This provides a controlled perturbation of local DNA mechanics without changing the underlying sequence or removing the phosphate group. We also generated matched libraries in which the terminal 5′ phosphate was removed, providing a complementary perturbation to identify positions where the phosphate itself contributes to TF binding. In both designs, the native base sequence is preserved. Libraries were printed on microarrays, incubated with TFs, and binding was quantified from fluorescence intensities, enabling position-resolved mapping of nick sensitivity across TF-binding sites (Materials and Methods; Extended Data Fig. 1). Signal intensities on DNA microarrays quantitatively reflect binding preferences and have previously been shown to correlate with *K*_D_ values measured by orthogonal methods^27–29^. For selected targets, we further measured thermodynamic and kinetic binding parameters directly. Thus, PIC-NIC provides a systematic, high-resolution framework for investigating how DNA backbone mechanics shapes TF binding across diverse structural families.

## PIC-NIC Reveals Position-Specific Nicking Sensitivity in 15 TFs

We applied PIC-NIC to a panel of 15 well-characterized TFs that bind sequence specifically, selected to span broad architectural diversity across eight DNA-binding families and diverse physiological roles (Extended Data Fig. 2). This panel includes CTCF, a central chromatin organizer; ETS1 and EGR1, extensively studied sequence-specific regulators with important roles in development, stress responses, and cancer; SOX2, a key pioneer factor involved in cell-fate control; TBP, a core component of the transcription machinery; MYC/MAX-family bHLH proteins, which include central oncogenic regulators; and bZIP factors such as CREB1 and ATF1, major signal-responsive transcriptional activators. For several families, the panel includes multiple members, enabling an initial assessment of within-family similarity in PIC-NIC sensitivity profiles.

For each TF, we introduced defined nicked constructs at every position across the binding motif, with either retention or removal of the 5′ phosphate (Extended Data Fig. 3; Supplementary Table 1). To systematically compare effects across proteins and positions, we quantified binding relative to the intact site and classified nick-induced changes into four categories: minimal (<10%), minor (10–50%), major (50–90%), and severe (>90%) loss of binding (Materials and Methods). Comparisons among families represented by multiple members suggested that features of PIC-NIC profiles are shared within some structural classes, including broad similarities among bHLH (Pearson’s r > 0.80 for any two members) and bZIP (Pearson’s r = 0.96) proteins, whereas zinc-finger proteins showed more variable profiles (Extended Data Fig. 3, 4a; Supplementary Table 1).

**Figure 2:**
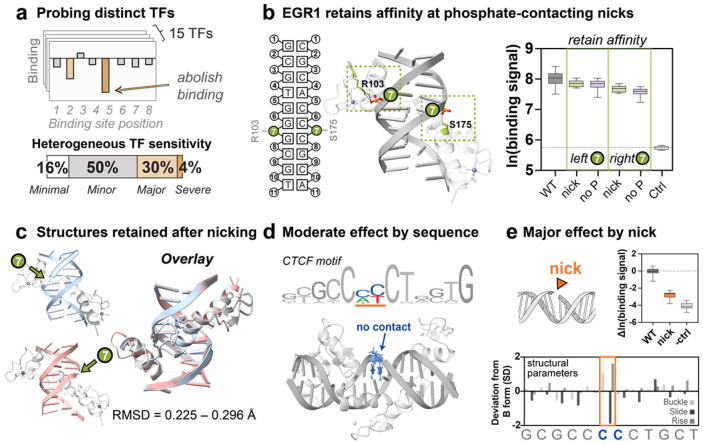
TFs exhibit heterogeneous sensitivity to DNA nicking. **(a)** Schematic illustration of a PIC-NIC binding profile (top), showing how binding is quantitatively measured at each nicked position relative to the intact control for individual TFs. Aggregate analysis across all 15 TFs (bottom; see Materials and Methods) classifies positions as retaining strong binding (<10% loss; white), minor disruption (10–50%; grey), major disruption (50–90%; gold), or abolishment of binding (>90%; brown), for nicks with 5′ phosphate retained. **(b)** *Left*: EGR1/DNA backbone phosphate contact map for both strands along the binding motif. Position 7 phosphates on both strands are in contact with the protein. *Right*: PIC-NIC binding profiles for EGR1 upon introducing site-specific nicks at positions 7 on both strands. Both left and right strand nicks at position 7 have little effect, indicating position-specific tolerance to backbone disruption. **(c)** High-resolution crystal structures of EGR1 bound to nicked DNA at position 7 on both strands (blue: left strand nick; pink: right strand nick), showing preservation of overall protein–DNA conformation despite the missing phosphate at the break site. Superposition of high-resolution crystal structures of EGR1 bound to intact DNA (gray) versus DNA nicked at phosphate position 7 on both strands (blue: left strand nick; pink: right strand nick). Minimal global structural deviation is observed (RMSD < 0.3 Å, overlay of all atoms), indicating high structural similarity between intact and nicked complexes. **(d)** For CTCF, mutating the two cytosines (CC) in the middle of the motif has a minor effect on the TF binding affinity, as visualized by the PWM logo (*top*), and the crystal structure (PDB ID: 5KKQ) shows that this site is a non-contact site (*bottom*). **(e)** PIC-NIC shows that introducing a nick between the CC base step markedly reduces binding affinity (*top*). The crystal structure shows that this site exhibits pronounced local DNA distortion, particularly increased buckle, slide, and rise (*bottom*). SD on the y-axis of the bar plot represents the standard deviation from the B-form envelope of DNA-protein complexes^29^.

Although one might expect a backbone break to induce substantial structural perturbations and broadly diminish TF binding, our measurements instead revealed a highly heterogeneous, position-specific response across motifs and TFs (Fig. 2a). A majority of positions tolerated backbone breaks and retained substantial binding upon nicking. Major disruption was observed at ~30% of positions, while severe loss of binding occurred at ~4% of positions, comparable to levels observed towards non-specific control sites.

Direct phosphate contact in the intact complex was not by itself sufficient to predict the response. Although nicking at some phosphate-contacting positions strongly disrupted binding, other contacted positions were unexpectedly tolerant. EGR1 illustrates this contrast. Several phosphate-contacting positions were sensitive to nicking, including two positions where the contacted phosphates are engaged by histidine residues that also coordinate zinc within the C2H2 motif (Extended Data Fig. 5a). Yet introducing a nick at position 7 on either strand, where the intact complex also contains phosphate contacts, had minimal effect on binding (Fig. 2b). Consistent with this tolerance, we solved two high-resolution crystal structures of EGR1 bound to DNA nicked at position 7 with the 5′ phosphate removed (PDB IDs: 9RIC, 9RJ6; Fig. 2c; Extended Data Table 2; Supplementary discussion). Both structures were nearly indistinguishable from the intact complex (PDB ID: 1AAY), with RMSD values of 0.225–0.296 Å (Fig. 2c). These findings demonstrate that even phosphate-contacting positions can, in some cases, accommodate backbone discontinuity and phosphate removal without substantial perturbation of the bound-state geometry. Thus, nicking does not universally weaken TF binding, even at apparent contact sites, and instead reveals position-specific tolerance that cannot be inferred from contact maps alone.

In contrast, the fraction of positions that exhibit strong sensitivity to nicking likely suggests that, in these cases, backbone integrity plays a mechanical role within the binding site. At these positions, even subtle disruption of strand continuity can markedly reduce binding despite preservation of base identity and, in the phosphate-retaining design, phosphate chemistry. This raises a key mechanistic question: what makes some nicking sites highly disruptive to TF–DNA complex stability, whereas others are functionally neutral? We therefore envisaged several mechanisms that may underlie this position-specific sensitivity to backbone discontinuity.

First, a nick could disrupt a mechanical anchor point where backbone continuity helps coordinate, directly or indirectly, multiple protein–DNA interactions^30,31^. Perturbing such an anchor, even locally, could alter the spatial arrangement required for complex stability and thereby produce a disproportionately large effect on binding.

Second, nick sensitivity may arise at positions that are deformed upon TF binding. Whereas base pairing and stacking help preserve structure in relaxed DNA^32,33^, positions subjected to TF-induced strain^34^ may depend more strongly on backbone continuity to maintain the bound geometry. A nick at such a site could locally relax the conformational constraint imposed by the intact backbone, preventing the DNA from achieving or retaining the strained conformation required for recognition (Fig. 1d). In these cases,the pronounced effect of nicking identifies positions where DNA mechanics itself becomes an active determinant of TF binding.

## Nick-Sensitive Sites Imply Structural Readout

If nick sensitivity reflects a dependence on strain-adapted DNA geometry, then nick-sensitive positions are expected to be enriched for local structural deviations in the corresponding TF–DNA complexes. To test this, we performed a structure-based analysis on all proteins in our PIC-NIC panel for which suitable high-resolution TF–DNA co-crystal structures were available (1.6 – 3.14 Å), thereby covering 13 of the 15 TFs analyzed.

We first focused on positions where neither the bases nor the flanking phosphates form hydrogen bonds with the protein, thereby minimizing contributions from direct protein–DNA contacts. Notably, 21% of these positions showed a significant reduction in binding upon nicking (Materials and Methods; Supplementary Tables 1–2). Structural analysis revealed that these nick-sensitive positions were enriched for deviations in base-step parameters, most prominently twist and buckle, compared with unaffected sites (Materials and Methods; Supplementary Table 2). To complement the structural analysis, we examined sequence-encoded DNA shape features using deepDNAshape^35^ and DNA intrinsic bendability^36,37^ across all TFs in our panel (Materials and Methods; Supplementary Tables 3-4). Of all positions and structural features tested with deepDNAshape, 4 passed the Bonferroni test, all of which were associated with nick-induced disruption of binding (at least a minor loss of binding after a nick) (Supplementary discussion).

These findings support a model in which binding at these positions depends on the ability of the DNA backbone to maintain a locally strained geometry. The enrichment of twist deviations is particularly consistent with the capacity of nicks to relax torsional constraint, as observed during topoisomerase-mediated DNA relaxation^38–42^.

Non-hydrogen-bonded, mechanically sensitive sites were observed across over a third of TFs in our study. Importantly, nick-sensitive sites do not consistently overlap with positions where base mutations strongly impair binding (Extended Data Fig. 3, Supplementary Tables 5-6). In some cases, nicks disrupt binding at positions largely unaffected by base substitutions, whereas in others, nicks have minimal impact even at positions highly sensitive to base identity.

For example, when probing the architectural factor CTCF, we found that nicking a specific position where base identity plays a relatively minor role resulted in one of the most pronounced reductions in binding (Fig. 2d–e). Analysis of the CTCF–DNA complex (PDB ID: 5KKQ) revealed that neither the bases nor the adjacent backbone phosphates at this position form direct hydrogen bonds with the protein (Fig. 2d), and the phosphate burial at this position is below average for the CTCF–DNA interface (Supplementary Table 7), consistent with limited dependence on base identity at this position. Yet the local base-step parameters at this position deviate sharply, with pronounced distortions in buckle, slide, and rise (Fig. 2e, bottom panel; Supplementary Table 2)^43^. These observations support a model in which impaired binding arises not from the loss of direct chemical readout, which is probed in studies relying on base mutations^44–48^, but from disrupting a strain-adapted DNA geometry that facilitates recognition.

To further dissect the mechanisms underlying nick sensitivity at sites lacking direct base contacts, we turned to ETS1, an oncogenic TF with extensive structural and biochemical characterization of its cognate DNA complexes^49^, including classic phosphate ethylation interference and missing-phosphate experiments^22,50^. ETS1 displayed sharply contrasting, position-dependent and phosphate-dependent responses to single-strand nicks: some sites tolerated backbone disruption with little or no effect, whereas others showed severe binding impairment or a moderate change that depended on whether the terminal phosphate was retained or removed.

**Figure 3:**
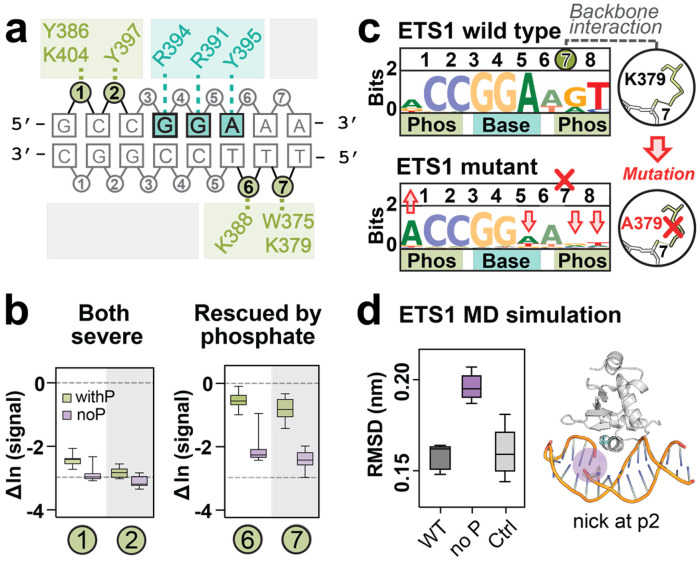
ETS1 demonstrates position-specific sensitivity to backbone continuity **(a)** ETS1 forms base-specific and phosphate-specific interactions across its recognition motif. Positions 1 & 2 on the top strand (left flank) and positions 6 & 7 on the bottom strand (right flank) are labeled as phosphate-contacting positions. Note, the sequence shown (5′ GCGGAAA 3′) and contacts obtained are from the crystal structure (PDB: 1K79), while the sequence used in PIC-NIC is the highest-affinity ETS1 binding site from our universal protein binding microarray data (5′ GCGGAAA 3′). **(b)** PIC-NIC phosphate removal profiles reveal that in the left flank, both phosphate-preserved nicks and phosphate-removed nicks strongly diminish ETS1 binding (*left*), while in the right flank, phosphate-removed nicks strongly disrupted binding and phosphate-preserved nicks rescued the binding (*right*). Y-axis denotes the difference in the natural log of the binding signal of the nicked binding site compared to the intact binding site, with 0 corresponding to the intact binding site. The lower dashed line denotes the level of non-specific binding. Each box represents 10 replicates of the same DNA binding site on the array. **(c)** Comparison of PWM logos for ETS1 wildtype protein and ETS1 mutant (K379A) protein. In the PWM logos, the y-axis displays information content in bits, and the numbers on the x-axis denote positions of backbone phosphates. When lysine 379 (WT protein), which interacts with backbone phosphate 7 in the ETS1 binding site, is substituted with an alanine (mutant protein), thus eliminating the H bond, a specificity change is observed. Red arrows in the PWM logos represent nucleotide positions where the most significant changes are observed after the amino acid mutation. **(d)** Representative snapshots of MD simulations (Materials and Methods) reveal reduced complex stability and greater structural disruption at the protein–DNA interface when a nick lacking the 5′ phosphate is introduced at position 2, as evidenced by elevated phosphate backbone RMSD values (5′ GCGGAAA 3′) compared to both the control nicked sequence and the wild-type duplex. Box plots display the average RMSD (compared to the initial frame) over the 1000 ns of simulation for five replicates per DNA/protein complex construct.

Introducing a nick and removing the 5′ phosphate within the GGA(A/T) core motif, where ETS1 forms direct hydrogen bonds with all three GGA bases (Fig. 3a)^51,52^, had only a minor effect on binding (Extended Data Fig. 3). In contrast, disrupting peripheral positions that contact phosphates produced a pronounced loss of binding (Fig. 3b, purple boxes), consistent with previous reports highlighting the importance of these phosphate interactions (Extended Data Fig. 6)^22,50^.

To further quantify this relationship, we analyzed the buried surface area (BSA) of phosphates in the ETS1–DNA complex. Across both strands, positions with more extensively buried phosphates showed stronger PIC-NIC sensitivity, indicating that these deeply embedded phosphate contacts are particularly vulnerable to disruption (Extended Data Fig. 7; Supplementary Table 7).

This distinction became apparent when we introduced phosphate-preserving nicks at the same peripheral sites (Fig. 3b, green boxes). At positions 6–7, retention of the 5′ phosphate largely rescued binding, suggesting that phosphate chemistry contributes directly to recognition at these sites. In contrast, positions 1–2 remained strongly disrupted despite retention of the phosphate, indicating that binding at these sites depends on backbone continuity or local geometry rather than phosphate chemistry alone. These contrasting outcomes reveal a functional dichotomy among phosphatecontacting positions that would be obscured by phosphate-removal or phosphate-interference assays alone.

If the right-flank phosphate contact contributes functionally to ETS1 recognition, then perturbing the residue that engages this phosphate provides an orthogonal way to test its role. Consistent with this interpretation, mutation of the key phosphate-contacting residue K379 shifted ETS1 base preferences across the binding site (Fig. 3c). Importantly, K379A did not become nonspecific, as it retained a coherent binding motif with clear information content at core positions (E-score ≥ 0.35^53^). Instead, its specificity was quantitatively reshaped: selectivity was reduced within the conserved GGA core and base preferences shifted at distal positions (Fig. 3c; Supplementary discussion; Extended Data Fig. 8; Supplementary Table 8). This effect may reflect direct coupling between phosphate contacts and base readout, consistent with these sites acting as anchoring points that help organize additional interactions. Alternatively, it could arise indirectly through allosteric rearrangements or altered domain flexibility, which in turn may modify the overall geometry of the binding interface.

We next focused on the left flank, where restoration of the phosphate did not rescue binding, and the nick alone was sufficient to abolish interaction. Bio-Layer Interferometry showed that a nick at position 2, with or without the 5′ phosphate, markedly accelerated ETS1 dissociation, reducing complex lifetimes to those of nonspecific DNA and effectively eliminating specific binding, whereas a control nick had minimal effect on kinetics (Extended Data Fig. 9, Supplementary Table 9). All-atom molecular dynamics simulations (Materials and Methods; Supplementary Table 10) further indicated that a nick at this position—which already exhibits pronounced twist and buckle deviations in the original PDB structure—induces substantial deformation, whereas the control nick largely preserves the bound-state geometry (Fig. 3d; Extended Data Fig. 10). These results support a model in which nicks introduced within strain-adapted regions produce amplified structural and energetic consequences.

These observations indicate that ETS1 relies on discrete mechanical anchors, in addition to chemical contacts, to direct DNA recognition. The contrasting behaviors on the two flanks are only fully observed using the PIC-NIC platform, which allows better decoupling of mechanical continuity and phosphate chemistry.

## Diverse Nick-Sensitivity Mechanisms in Deformed DNA Structures

Beyond simple reductions in binding affinity, we found that TF sensitivity to DNA nicks can manifest in diverse, position-specific ways. These effects include shifts in sequence specificity and even enhanced binding. To examine these effects mechanistically, we focused on TATA-binding protein (TBP) and SOX2, two well-characterized TFs known to induce substantial DNA deformation.

**Figure 4:**
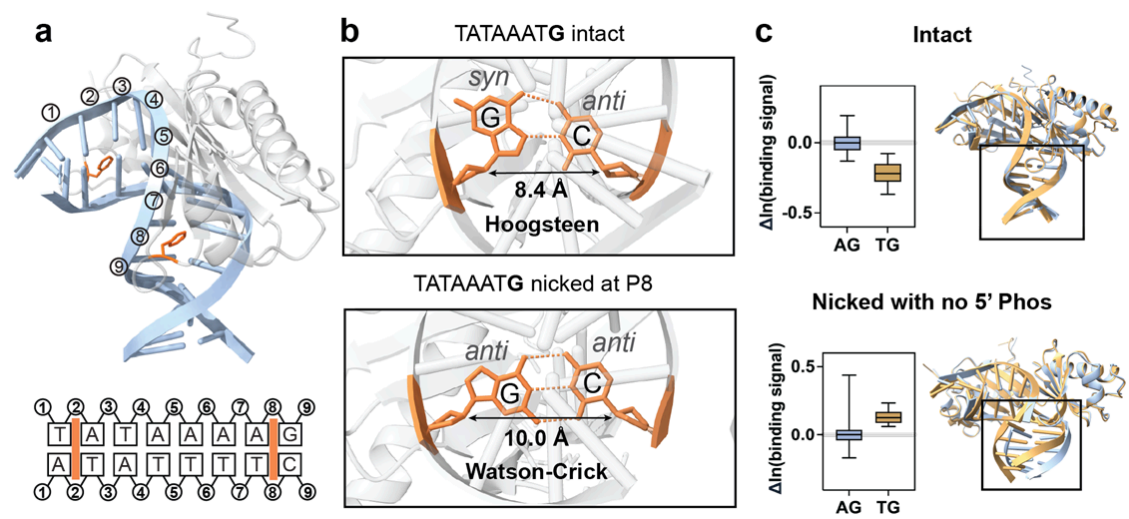
DNA nicks at Hoogsteen-prone positions reshape TBP sequence specificity. **(a)** The crystal structure of TBP bound to a bent TATA-box DNA (PDB ID:1QNE) shows key intercalation positions 2 and 8 (orange) by phenylalanine residues. **(b)** Close-up base pair geometry at position 8. In the intact TATAAATG complex (*top*), the terminal G–C of the binding site forms a Hoogsteen pair with an 8.4 Å C1′–C1′ distance. In the nicked complex (*bottom*), the same base pair adopts canonical Watson–Crick geometry with a widened 10.0 Å distance. **(c)** Difference in ln binding signal for TBP to two similar TATA-box variants: TATAAA**AG** (blue) and TATAAA**TG** (gold). In the intact duplex (*top*), TBP favors the AG variant, whilst upon nicking at position 8 on the bottom strand and removing the 5′ phosphate (*bottom*), preference switches to the TG variant. Structural overlays of the respective high-resolution crystal structures show high structural similarity in the case of intact DNA (RMSD = 0.190 Å). In the presence of the nick, increased bending in the nicked TG complex is observed (gold, RMSD = 0.465 Å; Extended Data Fig. 11).

TBP is a paradigmatic DNA-bending factor. It binds the minor groove of the TATA box and inserts two phenylalanine residues at positions 2 and 8, generating strong underwinding and an ~80° bend in the duplex^54^ (Fig. 4a). This creates a highly deformed, mechanically loaded DNA state. At the position 8 intercalation site, closely related sequence variants adopt distinct base-pair geometries in the TBP-bound complex. In the TATAAATG (“TG”) variant, the terminal G–C base pair adopts a Hoogsteen geometry, with guanine in the syn conformation, whereas in the TATAAAAG (“AG”) variant the corresponding base pair remains in a canonical Watson–Crick geometry^55^. In the intact duplex, TBP binds the AG variant more strongly than the TG variant, consistent with an energetic penalty associated with forcing the TG sequence into this strained Hoogsteen state^56–58^. These two related TATA-box variants therefore provide a sensitive system for testing whether local relaxation of backbone continuity at the intercalation site can alter DNA geometry and redirect TBP sequence preference. This interpretation is consistent with prior proposals that Hoogsteen base pairs can accommodate conformational stress in distorted DNA^58^, while suggesting that, in this TBP-bound context, adopting the Hoogsteen state imposes an energetic penalty that contributes to sequence preference.

To directly assess the contribution of DNA geometry at this position, we analyzed PIC-NIC binding signals for position 8 and determined four crystal structures of the corresponding nicked TBP–DNA complexes, with or without the 5′ phosphate (PDB IDs: **9OW8, 9OW7, 9OWZ and 9OWI**; Extended Data Fig. 11; Extended Data Table 3). Introducing a nick at position 8 in the TATAAATG complex shifted the terminal G–C pair from the strained Hoogsteen geometry observed in the intact complex to a canonical Watson–Crick geometry (Fig. 4b). In the phosphate-preserving nicked complex, this conformational transition occurs without changing the base sequence or removing phosphate chemistry, indicating that backbone continuity itself helps determine the DNA geometry adopted in the TBP-bound state.

This conformational shift was accompanied by a change in TBP binding preference. Whereas TBP favored TATAAAAG over TATAAATG in the intact duplex, nicking at position 8 shifted the relative preference toward TATAAATG (Fig. 4c). Thus, altering backbone continuity at a single mechanically sensitive position can change the relative affinity of two closely related sequences. In the phosphate-preserving design, this occurs without changing base identity or removing the terminal phosphate, indicating that the mechanical state accessible to the DNA can influence sequence recognition. These findings suggest that the energetic cost associated with the Hoogsteen geometry contributes to TBP specificity, and that nick-induced relaxation can reshape the preferred binding sequence.

**Figure 5:**
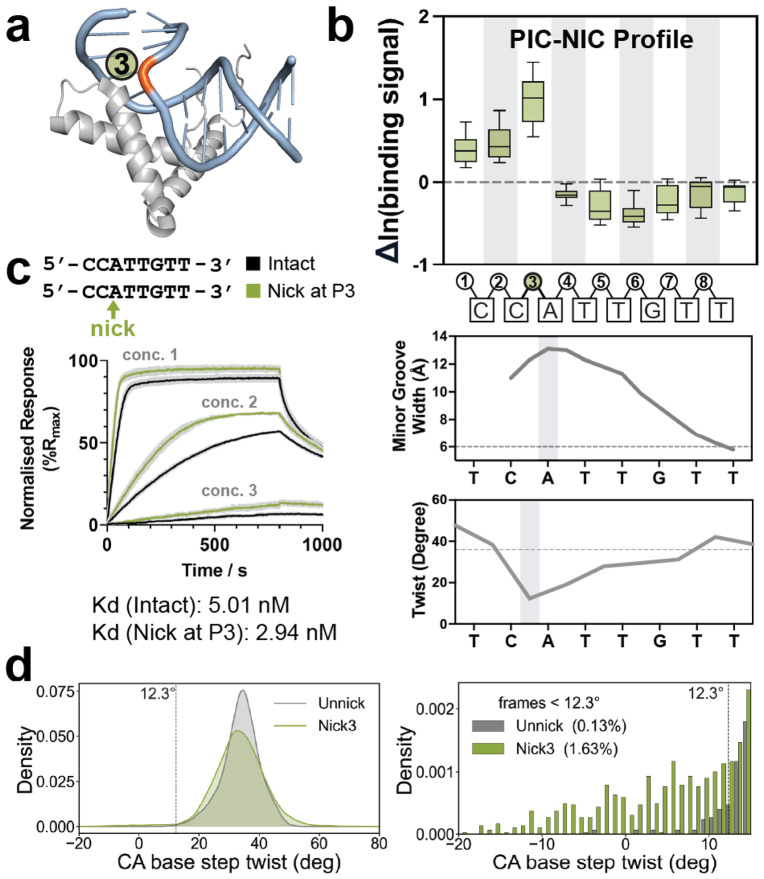
DNA nicks enhance SOX2 binding. **(a)** The crystal structure of SOX2 bound to DNA (PDB ID: 6HT5) shows that position 3 (orange) is highly kinked. **(b)** *Top panel*: PIC-NIC profile across the SOX2 motif reveals that a nick at position 3 significantly enhances the binding of SOX2. Dashed line represents the binding signal level of SOX2 to intact DNA. *Bottom panels*: SOX2 structure (PDB ID: 6HT5) reveals an extremely wide minor groove exceeding 13 Å and a significantly decreased twisting angle at this position. **(c)** Bio-Layer Interferometry (BLI) confirms enhanced binding upon nicking at position 3 (green), driven by faster association (*k*_on_). (**d**) MD simulations of SOX2–DNA complexes. Left: probability density of CA base-step twist angle for unnicked (grey) and nicked at position 3 (green) complexes. Dashed lines indicate twist value for the CA base-step in the intact bound crystal structure (12.3°). Right: zoom-in view of frames below 12.3°, showing ~12-fold enrichment in the nicked complex (1.63%) compared to unnicked control (0.13%).

TBP therefore illustrates how nick-mediated relaxation can rewire recognition by changing the conformational state of a strained base pair. We next asked whether nicking a mechanically strained site could also enhance binding rather than redirect specificity. SOX2 provides such an example. SOX2 binds in the minor groove and sharply bends its target DNA, typically by 70°–85^59,60^. The SOX2 complex therefore features DNA in a highly deformed state that requires substantial bending and twisting work from the protein (Fig. 5a). Within its motif, position 3 stands out as a key mechanical hotspot: the DNA is under-twisted and the minor groove width exceeds 13 Å (Fig. 5b, bottom panels)—more than double the ~6 Å width of canonical B-form DNA—marking a region where strain is strongly concentrated along the backbone.

PIC-NIC revealed that disrupting backbone continuity exactly at this hotspot enhances SOX2 binding. A phosphate-preserving nick at position 3 produced a ~3-fold increase in binding signal compared to the intact duplex (Fig. 5b, top panel), making it a unique case in our dataset where a nick strengthens, rather than weakens, TF–DNA recognition. These observations align with a model in which the nick partially offloads the mechanical penalty required to reach the SOX2-bound geometry: by releasing torsional constraint at the strained site, it may shift the ground-state ensemble toward conformations that are already closer to the distorted, recognition-competent state.

Kinetic measurements support this model. In contrast to ETS1, where a nick at a mechanical anchor primarily destabilized the complex by accelerating dissociation, the gain in SOX2 affinity was driven mainly by an increased association rate, with only modest changes in dissociation (Fig. 5c, Extended Data Fig. 9b-c). This behavior is expected if nicking enriches the pool of DNA molecules pre-biased toward the SOX2-favored bend and twist: SOX2 encounters more targets that are already mechanically primed, reducing the energy needed to deform the helix and enabling higher affinity.

Molecular dynamics simulations provide further mechanistic support for this model (Fig. 5d). In simulations of free DNA, we analyzed the CA base-step twist and compared the sampled conformations with the strongly undertwisted geometry observed in the SOX2-bound crystal structure (12.3°; compare to 36° for canonical B-form DNA). Nicking at position 3 markedly enriched conformations approaching this bound-state geometry, which were sampled in 1.63% of frames compared with 0.13% in the unnicked control, corresponding to an ~12-fold enrichment. These results suggest that the nick locally releases torsional constraints and shifts the ground-state DNA ensemble toward conformations that are mechanically primed for SOX2 recognition. Complementary hydrogen-bond analysis of simulations of the bound complex further suggests that nicking may also enable additional stabilizing contacts (Extended Data Fig. 12).

Past studies have demonstrated similar effects for other DNA-bending proteins^14,24^, showing that nicked or gapped sites can enhance binding by reducing the energetic cost of DNA deformation. Together, these results also raise the broader possibility that other TFs—particularly those acting within compacted or topologically constrained chromatin—may similarly respond to structural perturbations that promote their bound DNA geometries. The structurally sensitive positions identified here may represent sites where deformations—such as those imposed by nucleosomes or partner TFs^8,10,61–63^—modulate TF binding without altering the underlying sequence.

Together, both the heterogeneous global landscape and the specific examples of sensitivity to backbone continuity observed in our study provide a compelling indication that not all contacting positions across TF binding sites are mechanically or energetically equivalent. Some examples—such as EGR1 position 7—are structurally and functionally buffered: their removal produces only modest changes in affinity and leaves the bound conformation essentially unchanged. On the other hand, different examples indicate mechanical anchors in the backbone (ETS1, position 2) or strain hotspots whose perturbation rewires recognition (TBP, position 8) or even enhances binding affinity. PIC-NIC thus distinguishes, at nucleotide resolution, backbone linkages that participate in strain-adapted recognition from those that are mechanically neutral.

## Exploring the Relationship Between TF-bound Nicks and DNA Repair

PIC-NIC uses site-specific nicks as controlled mechanical perturbations *in vitro*, but backbone discontinuities are also physiologically relevant DNA lesions. SSBs are among the most common forms of DNA damage^64^, and recent studies suggest that proteins bound at damaged DNA can restrict access by repair machinery^65,66^. This raises two related questions: do SSBs accumulate within TF-occupied regulatory regions, and, if so, can TFs that remain bound to nicked sites interfere with repair-factor access or repair synthesis?

To address these questions, we analyzed available genome-wide datasets from induced neurons (iNeurons), including SSB maps generated by S1-END-seq, signals associated with SSB repair signaling and recruitment measured by poly(ADP-ribose) (PAR) and XRCC1 ChIP-seq, and repair synthesis activity measured by SAR-seq^67^. We focused on EGR1 and CTCF, two TFs characterized by PIC-NIC in this study and with established roles in neuronal function^68,69^.

**Figure 6:**
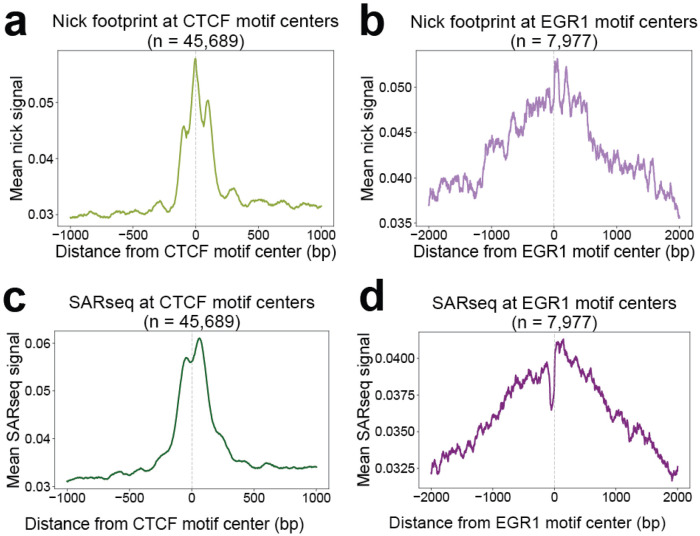
SSBs accumulate at TF binding sites and repair synthesis is locally impaired in vivo. **(a-b)** **Aggregate** S1-END-seq signal (mean nick signal, ±95% CI) centered on CTCF (**a**, n = 45,689) and EGR1 (**b**, n = 7,977) binding motifs in iNeurons, showing enrichment of single-strand breaks at TF motif centers. Dashed line indicates motif center. **(c-d)** Aggregate SAR-seq signal (mean repair synthesis signal, ±95% CI) at CTCF (**c**) and EGR1 (**d**) motif centers. A local depletion of repair synthesis signal is observed directly at the motif center, flanked by elevated signal on both sides.

S1-END-seq maps revealed significant enrichment of SSBs at the centers of EGR1 and CTCF ChIP-seq peaks containing the corresponding TF motifs (Fig. 6a–b). This suggests that SSB accumulation occurs at occupied TF-binding sites, consistent with previous reports showing that regulatory regions, including promoters and enhancers, are enriched for DNA breaks^67^.

SAR-seq revealed elevated repair synthesis around these TF-binding sites, consistent with increased repair activity near regions of SSB accumulation. However, this signal showed a significant local minimum directly at the motif center (Extended Data Fig. 13 d, h; Supplementary discussion), flanked by elevated repair synthesis on both sides (Fig. 6c,d). Similar repair profiles, with local depletion at TF-bound sites, have been reported for nucleotide excision repair and interpreted as evidence that bound TFs can impede repair locally^66^. PAR and XRCC1 ChIP-seq signals were also enriched around these TF-binding sites, consistent with engagement of the SSB repair pathway. Notably, PAR signal also showed a local depletion at the motif center, further suggesting that TF occupancy may restrict repair-factor access or activity at the immediate binding site (Extended Data Fig. 13; Supplementary discussion). These analyses establish a broad association between TF occupancy and altered SSB repair activity, but do not yet resolve whether specific within-motif nick positions differentially influence repair outcomes as SAR-seq is currently limited to ~150-200 nt resolution. Directly testing PIC-NIC’s position-specific predictions *in vivo* remains an exciting direction as higher-resolution repair maps emerge.

To test in a controlled setting whether TF occupancy can limit repair-factor access to nicked DNA, we performed targeted PIC-NIC competition experiments in a cell-free, on-chip format. Motivated by recent studies showing that DNA-bound proteins can restrict access of repair factors to damaged DNA^65^ and past experiments demonstrating that proteins can compete with repair and processing enzymes for occupancy at nicked DNA sites^70^, we examined competition between ETS1 and PARP1 at three nicked DNA sites. ETS1 was selected because it is a well-established model for TF–DNA damage interactions and repair-factor competition^66,71–74^, and because its PIC-NIC profile includes both nick-sensitive and nick-tolerant positions (Materials and Methods).

These experiments revealed an overall reduction in PARP1 binding in the presence of ETS1, consistent with competition between TF occupancy and repair-factor access (Extended Data Fig. 7b; Supplementary Table 12). However, the extent of PARP1 displacement differed among nicked sites. PARP1 binding was reduced most strongly at the nicked site where ETS1 occupancy was highest, whereas a site where ETS1 binding was substantially disrupted retained greater PARP1 accessibility (Extended Data Fig. 7c-d). This pattern supports the idea that TF retention at nicked DNA can restrict repair-factor binding. However, a second nicked site with strong ETS1 binding did not show the same degree of PARP1 displacement, indicating that repair-factor access is not determined by TF occupancy alone (Extended Data Fig. 7c-d; Supplementary discussion). Thus, TF-mediated interference with PARP1 binding is position-dependent and likely depends not only on TF occupancy, but also on the local DNA structure, protein–DNA geometry, and PARP1 recognition context.

Together, these genomic analyses and cell-free competition experiments support a model in which TF retention at nicked DNA can reduce repair-factor accessibility in a position-dependent manner. PIC-NIC therefore provides a framework for identifying TF-bound breaks that are likely to remain exposed to repair factors versus those that may be partially occluded within stable TF–DNA complexes.

## Discussion

Our study reveals that DNA backbone integrity plays an active and position-specific role in TF recognition. By systematically introducing site-specific single-strand breaks, we move towards decoupling DNA backbone integrity from base identity and understanding how disruptions in DNA geometry reshape TF binding affinity, specificity, and kinetics across 15 human TFs spanning diverse structural classes. This decoupling exposes an often-hidden layer of recognition logic encoded not in the linear sequence but in the mechanical and geometric architecture of the DNA helix.

We find that discrete backbone positions serve as structural anchor points, where even subtle disruptions destabilize binding (e.g. ETS1), rewire sequence preferences (e.g. TBP), or in some cases, enhance affinity by reshaping DNA conformation (e.g. SOX2). Notably, these structurally sensitive sites often lie outside the defined base core sequence. These findings challenge the conventional base-centric model of TF recognition and provide new insights into the elusive role of the DNA backbone.

Several additional observations reinforce and extend this picture. Across the bHLH family, deepDNAshape analysis revealed that predicted roll or rise at the central CG palindrome step independently emerges as the strongest correlate of binding preference across four members—precisely the position where PIC-NIC identifies the greatest nick sensitivity—suggesting that this could be the mechanical hotspot of the bHLH family proteins (Supplementary discussion; Extended Data Fig. 4b-c; Supplementary Table 3). Phosphate burial analysis similarly revealed position-specific trends for some proteins: strong correlations between burial depth and nick sensitivity were observed for ETS1 and FOXA2 (Supplementary Table 7; Supplementary discussion), but not consistently across the full panel, indicating that contact surface area captures part but not all of the structural logic underlying backbone-mediated recognition. Finally, the contrasting kinetic responses observed for ETS1 and SOX2 point toward a broader potential mechanistic framework: positions where backbone disruption accelerates dissociation may reflect structural anchor points whose loss destabilizes an assembled complex, while positions where disruption enhances association may reflect strain hotspots whose relaxation lowers the barrier to reaching the bound geometry. Systematic kinetic characterization across the PIC-NIC dataset could test this framework more broadly and begin to establish predictive relationships between local DNA architecture and binding mechanism.

Beyond single-strand breaks, additional physiological DNA processes—including transcription-induced supercoiling, nucleosome remodeling, chromatin compaction, and binding of other transcription factors—continuously generate local mechanical distortions that reshape DNA conformation without altering base sequence. More broadly, our findings raise the possibility that, much like the effects revealed by PIC-NIC, such structural fluctuations may fine-tune TF binding affinity and specificity *in vivo*, depending on whether they perturb structurally sensitive anchor points. While many deformations are likely neutral, disruptions at these key positions could produce pronounced shifts in TF binding behavior and regulatory output. This adds a dynamic, context-dependent structural layer to transcriptional regulation, where DNA architecture shaped by multiple factors continuously modulates gene expression.

Extending these measurements to chromatinized DNA would provide a way to examine additional layers of backbone-sensitive recognition in a more physiologically relevant structural context. Nucleosomes impose defined bending, twisting, and rotational constraints on DNA, while also modulating accessibility through wrapping and transient unwrapping. Applying PIC-NIC-like perturbations to *in vitro* reconstituted nucleosomes^75,76^ would create a controlled framework to test whether backbone-sensitive positions identified on naked DNA remain functionally relevant under chromatin constraints, and whether additional sensitivities emerge from the interplay between TF binding and nucleosome architecture. Such experiments could distinguish cases in which sensitivity to backbone disruption reflects intrinsic features of the local TF–DNA interface from those in which sensitivity is reshaped by chromatin architecture. More broadly, this approach would help bridge the gap between *in vitro* measurements and the *in vivo* environment, where DNA sequence, backbone continuity, nucleosome positioning, accessibility, and local mechanical constraints are integrated to govern TF binding (Supplementary discussion).

Importantly, our findings suggest a structural framework for how DNA damage may intersect with both gene regulation and genome stability. Single-strand breaks—the most frequent form of DNA damage^64^— may displace or retain TFs depending on the position, altering transcriptional programs in a position-dependent manner that remains to be fully understood.

Beyond transcriptional modulation, growing evidence suggests that TF binding to damaged DNA can actively interfere with the repair process, reducing repair efficiency and elevating mutational risks. Conversely, some TFs may stabilize lesion recognition and facilitate repair initiation^65,66,77–79^. In a recent study, TF binding was shown to directly compete with mismatch repair machinery, with TFs outcompeting MutSα for occupancy at replication error sites and thereby elevating mutagenesis at TF binding sites in both yeast and human cancers^65^. By systematically charting how backbone discontinuities reshape TF binding at nucleotide resolution, PIC-NIC suggests which lesions will release TFs and open DNA for repair, and which will retain TF occupancy, rendering repair more challenging. This framework provides a stepping stone to connect the structural logic of TF recognition to repair vulnerability, offering predictive insight into genome stability and mutagenesis.

Together, our findings shed light on a subtle yet significant mechanism at play in DNA/protein recognition that has been challenging to fully understand with classical approaches. Beyond the one-dimensional sequence code, we uncover a structural recognition layer embedded in the DNA backbone—a dynamic mechanical grammar shaped by both evolutionary constraints and transient cellular forces, expanding the regulatory logic that governs transcriptional control, genome maintenance, and cellular identity. The PIC-NIC platform can be readily extended to a broader repertoire of transcription factors, offering a scalable framework for systematically dissecting how DNA backbone architecture shapes specificity across diverse regulatory contexts. By illuminating a structural recognition layer that has long been challenging to deconvolute, PIC-NIC offers new opportunities to understand the mechanical nature of protein-DNA recognition.

## Methods

### Protein expression and purification

For PIC-NIC experiments, full-length human ETS1, human EGR1 DNA-binding domain (residues 335-423), full-length *Arabidopsis thaliana* TBP, full-length human RUNX1 and human SOX2 DNA-binding domain (residues 19-118) were expressed and purified as described in Supplementary Methods. Full-length human Max and DNA-binding domain of Myc, Mad and Mnt were chemically synthesized as described previously^80,81^. Human FOXA2 DNA-binding domain (residues 142-269) was expressed by *in vitro* transcription/translation system with PURExpress^®^ *In Vitro* Protein Synthesis Kit (Supplementary Methods)^82^. Full-length human MITF, CREB1, ATF1, SP1 and CTCF were obtained commercially (Supplementary Methods). For ETS1 BLI experiments, murine ETS1 (residues 331-440) was produced as in Supplementary Methods. For SOX2 BLI experiments, the same proteins as in PIC-NIC experiments were used. For EGR1 and TBP X-ray crystallization, the same proteins were used as described above, but with the tag cleaved (Supplementary Methods).

### Universal Protein Binding Microarray

Transcription factor (TF) binding characterization was performed using universal protein binding microarrays (PBMs), as described previously^83,84^. Briefly, commercial microarrays (Agilent Technologies) containing all possible 10-mer or 9-mer sequences were converted to double-stranded DNA via solid-phase primer extension using Thermo Sequenase DNA Polymerase (Cytiva, Catalog #: E79000Y) and a deoxynucleotide triphosphate (dNTP) mixture (dATP, dCTP, dGTP, dTTP). Microarrays were then blocked with 2% (w/v) non-fat dry milk (Sigma, Catalog #: M7409) and incubated with the TF of interest. Binding reactions were carried out in protein-specific buffers (Supplementary Methods).

Pre-incubated protein binding mixtures were applied to individual microarray chambers, incubated for 1 h at room temperature, then subjected to two sequential washing steps. Next, microarrays were incubated for 1 h at room temperature with fluorescently labelled antibody diluted in protein binding buffer supplemented with 2% milk, according to the epitope tag of the protein. Following antibody incubation, microarrays were subjected to two sequential washing steps (Supplementary Methods). Fluorescence signals of the bound proteins were recorded using a GenePix^®^ 4400A scanner. Signal intensities were extracted using GenePix Pro 7.0 software and median pixel intensity was reported for each DNA probe, before further analysis (Supplementary Methods).

### PIC-NIC library design and measurements

PIC-NIC experiments were performed as follows. Libraries of DNA complexes with a nick at every possible position in the TF binding site were designed for each TF, with and without the phosphate at 5′ end of the breakage site. Nick DNA complexes were constructed in two ways. In the first method, a single-stranded oligo was designed to fold on itself upon annealing, forming a dumbbell-shaped DNA, with a nick at the desired position and an amino modifier on the loop of the dumbbell. In the second method, three strands of oligos were designed to anneal and form a duplex, with the two shorter strands being the complements of the longer strand, leaving a nick at the position desired (Extended Data Fig. 1a). The longer strand is labelled with an amino modifier. In the first method, the negative control sequence contained a nick remote from the TF binding site; in the second method, the negative control sequence was the corresponding intact double-stranded duplex.

For each probe, thermodynamic parameters of hybridization were evaluated using the web-based tools from Integrated DNA Technologies and NUPACK, to verify the integrity of annealing under experimental conditions. Simulations incorporated strand concentrations and ionic conditions mimicking experimental settings (150 mM Na^+^, 5 mM Mg^2+^), with appropriate ion correction terms. The maximum complex size was set to 2 for intact duplexes and 3 for nicked complexes involving three strands.

The libraries were spotted onto epoxy-functionalized glass slides using a sciFLEXARRAYER S12 automated non-contact dispensing system (Scienion), followed by rehydration and blocking steps to yield PIC-NIC chips (Supplementary Methods). Protein binding experiments on PIC-NIC chips were performed using the same conditions as universal PBMs (Supplementary Methods). The chips were scanned and the binding signal intensities were recorded the same way as universal PBMs above.

After extracting the measured probe intensities, we quantified the extent of binding disruption at each site by first taking the natural logarithm (ln) of the fluorescence signal. For each nicked site, we computed the percentage signal change relative to an intact control binding site (Supplementary Methods). This was done by calculating the normalized signal change: Signal change = [ln(*I*_intact_) – ln(*I*_non-binding_)] / [ln(*I*_intact_) – ln(*I*_nicked_)], where *I*_intact_ is the signal from an intact binding site, *I*_non binding_is the signal from a non-binding site, and *I*_nicked_ is the signal from the desired nicked site. Sites were then classified into four categories: retention of binding (<10% decrease in signal), minor disruption (10–50%), major disruption (50–90%), and abolishment of binding (>90%).

### Site-Directed Mutagenesis

Site-directed mutagenesis was performed using KAPA HiFi HotStart DNA Polymerase (Roche) according to the manufacturer’s standard protocol. Mutagenic primers were designed (Supplementary Methods) to introduce the desired substitution. Following PCR amplification, the reaction was treated with DpnI (NEB) to digest the template plasmid, and the product was transformed into *E. coli* DH5α. Positive clones were confirmed by miniprep and Sanger sequencing.

### Crystallization and determination of the structure of TBP-nicked DNA and EGR1-nicked DNA complexes

TBP–DNA complexes were prepared and subjected to hanging drop vapor diffusion crystallization screens (Supplementary Methods), resulting in large, well-diffracting crystals suitable for data collection after optimization of initial hits. Data for all crystals were collected at the Advanced Light Source (ALS) on beamlines 5.0.1 and 5.0.2. The data were processed with XDS^85^. The structures were solved by molecular replacement (MR) using a previous structure of TBP (Supplementary Methods) as a search model. MolProbity was used to guide the process of refitting and refinement^86^. See Extended Data Table 1 for the final data collection and refinement statistics for each structure.

EGR-DNA complexes were prepared as previously described^15^ and subjected to hanging drop vapor diffusion crystallization screens (Supplementary Methods), yielding large and well-diffracting crystals suitable for data collection in the initial screens. Data for all the crystals were collected at The Dana and Yossie Hollander Center for Structural Proteomics at the Weizmann Institute of Science. Initial models were iteratively rebuilt and refined using Coot^87^ and Phenix^88^. Model geometry was evaluated using MolProbity^86^. See Extended Data Table 2 for the final data collection and refinement statistics for each structure.

### All-atom molecular dynamics simulation

All-atom MD simulations were performed for ETS1 and SOX2 systems using GROMACS 2024.2. For ETS1, nicked and unnicked DNA–protein complexes were constructed from PDB ID 1K79 (DNA sequence 5′ TAGTGCCGGAAATGT 3′), with nicks introduced at position 7 on either strand using PyMOL. For SOX2, systems were constructed from PDB ID 6HT5 (DNA sequence 5′ CCCCATTGTTATGC 3′), with nicks introduced at positions C4 or T7. All proteins were simulated with Amberff19sb^89^ and all DNA were simulated with OL15^90^ forcefield. All systems were solvated with TIP3P water model and neutralized with 0.15 M KCl using CHARMM-GUI Solution Builder^91^. Systems were energy minimized, equilibrated for 10 ns with positional restraints on heavy atoms, and then simulated for 1 μs in triplicate with 2 fs timesteps at 310 K and 1 bar. Hydrogen bond analyses were performed using GROMACS 2024.2. Full simulation parameters and box compositions are provided in Supplementary Methods and Supplementary Table 10.

### Genome-wide analysis of single-strand breaks and repair signals at transcription factor binding sites

EGR1 and CTCF ChIP-seq peaks were obtained from ENCODE (accessions: ENCFF016RNL and ENCFF930BXV, respectively). Genome-wide SSB data were obtained from Wu et al.^67^ (S1-END-seq, GSM5100382), alongside PAR ChIP-seq (GSM5100373), XRCC1 ChIP-seq (GSM5100374), and SAR-seq (GSM5100400-402) from induced neurons (iNeurons). All datasets were mapped to hg19 using UCSC liftOver; ENCODE blacklist^92^ regions were excluded throughout. Genome-wide EGR1 (MA0162.5) and CTCF (MA0139.2) motif matches were obtained from JASPAR^93^. For each TF, aggregate signal profiles were computed by centering on ChIP-seq peak midpoints and on the highest-scoring motif within each peak. Nick enrichment at TF binding sites was assessed by permutation testing against 1,000 sets of size-matched random genomic regions, with fold enrichment, Z-scores, and empirical p-values reported. SAR-seq peaks were called using a custom percentile-threshold approach retaining the top 1% of genome-wide signal. Full details of data acquisition, coordinate conversion, signal quantification, motif-centered analysis, enrichment testing, and peak calling are provided in Supplementary Methods.

## Supplementary Material

This is a list of supplementary files associated with this preprint. Click to download.

- NSMBSupplementaryMaterials.pdf
- NSMBExtendedDataFiguresandTables.pdf
- SupplementaryTables.zip

## Data Availability

The data that support the findings in this study are available as Supplementary Tables in Excel format. Coordinates and structure factor amplitudes for the TBP-AG-withP, TBP-AG-noP, TBP-TG-withP, TBP-TG-noP, EGR-n7, EGR-ren5P and EGR-ren7 structures have been deposited in the PDB under the accession codes 9OWZ, 9OWI, 9OW8, 9OW7, 9RIC, 9RI6, and 9RJ6, respectively. The PDB entries used in this study are available in Extended Data Figs. 2 and Supplementary Tables 2. Molecular dynamics simulations tpr, mdp and itp files are stored at doi:10.5281/zenodo.15646043 and 10.5281/zenodo.15646042.

## References

[1] Kim S. & Shendure J. Mechanisms of Interplay between Transcription Factors and the 3D Genome. Mol Cell 76, 306–319 (2019). 10.1016/j.molcel.2019.08.010 31521504

[2] Garvie C. W. & Wolberger C. Recognition of specific DNA sequences. Mol Cell 8, 937–946 (2001). 10.1016/s1097-2765(01)00392-6 11741530

[3] Basu A. Measuring DNA mechanics on the genome scale. Nature 589, 462–467 (2021). 10.1038/s41586-020-03052-3 33328628 PMC7855230

[4] Rohs R. Origins of specificity in protein-DNA recognition. Annu Rev Biochem 79, 233–269 (2010). 10.1146/annurev-biochem-060408-091030 20334529 PMC3285485

[5] Rohs R. The role of DNA shape in protein-DNA recognition. Nature 461, 1248–1253 (2009). 10.1038/nature08473 19865164 PMC2793086

[6] Erie D. A., Yang G., Schultz H. C. & Bustamante C. DNA bending by Cro protein in specific and nonspecific complexes: implications for protein site recognition and specificity. Science 266, 1562–1566 (1994). 10.1126/science.7985026 7985026

[7] Mathelier A. DNA Shape Features Improve Transcription Factor Binding Site Predictions In Vivo. Cell Syst 3, 278–286 e274 (2016). 10.1016/j.cels.2016.07.001 27546793 PMC5042832

[8] Dodonova S. O., Zhu F., Dienemann C., Taipale J. & Cramer P. Nucleosome-bound SOX2 and SOX11 structures elucidate pioneer factor function. Nature 580, 669–672 (2020). 10.1038/s41586-020-2195-y 32350470

[9] Mitra J. Extreme mechanical diversity of human telomeric DNA revealed by fluorescence-force spectroscopy. Proc Natl Acad Sci U S A 116, 8350–8359 (2019). 10.1073/pnas.1815162116 30944218 PMC6486787

[10] Mariani L. DNA bendability regulates transcription factor binding to nucleosomes. Nat Struct Mol Biol 32, 2185–2195 (2025). 10.1038/s41594-025-01633-2 40855132 PMC12884659

[11] Basu A. Deciphering the mechanical code of the genome and epigenome. Nat Struct Mol Biol 1178–1187 (2022). 10.1038/s41594-022-00877-6 36471057 PMC10142808

[12] Harrington R. E. DNA curving and bending in protein-DNA recognition. Mol Microbiol 6, 2549–2555 (1992). 10.1111/j.1365-2958.1992.tb01431.x 1333034

[13] Kim Y., Geiger J. H., Hahn S. & Sigler P. B. Crystal structure of a yeast TBP/TATA-box complex. Nature 365, 512–520 (1993). 10.1038/365512a0 8413604

[14] Schreck R., Zorbas H., Winnacker E. L. & Baeuerle P. A. The NF-kappa B transcription factor induces DNA bending which is modulated by its 65-kD subunit. Nucleic Acids Res 18, 6497–6502 (1990). 10.1093/nar/18.22.6497 2174540 PMC332601

[15] Pavletich N. P. & Pabo C. O. Zinc finger-DNA recognition: crystal structure of a Zif268-DNA complex at 2.1 A. Science 252, 809–817 (1991). 10.1126/science.2028256 2028256

[16] Mulero M. C. DNA-binding affinity and transcriptional activity of the RelA homodimer of nuclear factor kappaB are not correlated. J Biol Chem 292, 18821–18830 (2017). 10.1074/jbc.M117.813980 28935669 PMC5704467

[17] Piper D. E., Batchelor A. H., Chang C. P., Cleary M. L. & Wolberger C. Structure of a HoxB1-Pbx1 heterodimer bound to DNA: role of the hexapeptide and a fourth homeodomain helix in complex formation. Cell 96, 587–597 (1999). 10.1016/s0092-8674(00)80662-5 10052460

[18] Bushman F. D., Anderson J. E., Harrison S. C. & Ptashne M. Ethylation interference and X-ray crystallography identify similar interactions between 434 repressor and operator. Nature 316, 651–653 (1985). 10.1038/316651a0 4033762

[19] Brunelle A. & Schleif R. F. Missing contact probing of DNA-protein interactions. Proc Natl Acad Sci U S A 84, 6673–6676 (1987). 10.1073/pnas.84.19.6673 2958845 PMC299145

[20] Hayes J. J. & Tullius T. D. The missing nucleoside experiment: a new technique to study recognition of DNA by protein. Biochemistry 28, 9521–9527 (1989). 10.1021/bi00450a041 2611245

[21] Mohibullah N., Donner A., Ippolito J. A. & Williams T. SELEX and missing phosphate contact analyses reveal flexibility within the AP-2[alpha] protein: DNA binding complex. Nucleic Acids Res 27, 2760–2769 (1999). 10.1093/nar/27.13.2760 10373594 PMC148486

[22] Wang H., McIntosh L. P. & Graves B. J. Inhibitory module of Ets-1 allosterically regulates DNA binding through a dipole-facilitated phosphate contact. J Biol Chem 277, 2225–2233 (2002). 10.1074/jbc.M109430200 11689571

[23] Okonogi T. M., Alley S. C., Harwood E. A., Hopkins P. B. & Robinson B. H. Phosphate backbone neutralization increases duplex DNA flexibility: a model for protein binding. Proc Natl Acad Sci U S A 99, 4156–4160 (2002). 10.1073/pnas.072067799 11929991 PMC123618

[24] Kim S. S., Tam J. K., Wang A. F. & Hegde R. S. The structural basis of DNA target discrimination by papillomavirus E2 proteins. J Biol Chem 275, 31245–31254 (2000). 10.1074/jbc.M004541200 10906136

[25] Jen-Jacobson L. Protein-DNA recognition complexes: conservation of structure and binding energy in the transition state. Biopolymers 44, 153–180 (1997). 10.1002/(SICI)1097-0282(1997)44:2<153::AID-BIP4>3.0.CO;2-U 9354759

[26] Malygin E. G., Petrov N. A., Gorbunov Y. A., Kossykh V. G. & Hattman S. Interaction of the phage T4 Dam DNA-[N6-adenine] methyltransferase with oligonucleotides containing native or modified (defective) recognition sites. Nucleic Acids Res 25, 4393–4399 (1997). 10.1093/nar/25.21.4393 9336474 PMC147042

[27] Siggers T., Duyzend M. H., Reddy J., Khan S. & Bulyk M. L. Non-DNA-binding cofactors enhance DNA-binding specificity of a transcriptional regulatory complex. Mol Syst Biol 7, 555 (2011). 10.1038/msb.2011.89 22146299 PMC3737730

[28] Carlson C. D. Specificity landscapes of DNA binding molecules elucidate biological function. Proc Natl Acad Sci U S A 107, 4544–4549 (2010). 10.1073/pnas.0914023107 20176964 PMC2842033

[29] Afek A. DNA mismatches reveal conformational penalties in protein-DNA recognition. Nature 587, 291–296 (2020). 10.1038/s41586-020-2843-2 33087930 PMC7666076

[30] Luscombe N. M., Laskowski R. A. & Thornton J. M. Amino acid-base interactions: a three-dimensional analysis of protein-DNA interactions at an atomic level. Nucleic Acids Res 29, 2860–2874 (2001). 10.1093/nar/29.13.2860 11433033 PMC55782

[31] Hancock S. P., Cascio D. & Johnson R. C. Cooperative DNA binding by proteins through DNA shape complementarity. Nucleic Acids Res 47, 8874–8887 (2019). 10.1093/nar/gkz642 31616952 PMC7145599

[32] Yakovchuk P., Protozanova E. & Frank-Kamenetskii M. D. Base-stacking and base-pairing contributions into thermal stability of the DNA double helix. Nucleic Acids Res 34, 564–574 (2006). 10.1093/nar/gkj454 16449200 PMC1360284

[33] Hunter C. A. Sequence-dependent DNA structure. The role of base stacking interactions. J Mol Biol 230, 1025–1054 (1993). 10.1006/jmbi.1993.1217 8478917

[34] Nelson P. Transport of torsional stress in DNA. Proc Natl Acad Sci U S A 96, 14342–14347 (1999). 10.1073/pnas.96.25.14342 10588707 PMC24438

[35] Li J. & Rohs R. Deep DNAshape webserver: prediction and real-time visualization of DNA shape considering extended k-mers. Nucleic Acids Res 52, W7–W12 (2024). 10.1093/nar/gkae433 38801070 PMC11223853

[36] Brukner I., Sanchez R., Suck D. & Pongor S. Sequence-dependent bending propensity of DNA as revealed by DNase I: parameters for trinucleotides. EMBO J 14, 1812–1818 (1995). 10.1002/j.1460-2075.1995.tb07169.x 7737131 PMC398274

[37] Jiang W. J. Assessing base-resolution DNA mechanics on the genome scale. Nucleic Acids Res 51, 9552–9566 (2023). 10.1093/nar/gkad720 37697433 PMC10570052

[38] Pommier Y., Sun Y., Huang S. N. & Nitiss J. L. Roles of eukaryotic topoisomerases in transcription, replication and genomic stability. Nat Rev Mol Cell Biol 17, 703–721 (2016). 10.1038/nrm.2016.111 27649880 PMC9248348

[39] Champoux J. J. DNA topoisomerases: structure, function, and mechanism. Annu Rev Biochem 70, 369–413 (2001). 10.1146/annurev.biochem.70.1.369 11395412

[40] Koster D. A., Croquette V., Dekker C., Shuman S. & Dekker N. H. Friction and torque govern the relaxation of DNA supercoils by eukaryotic topoisomerase IB. Nature 434, 671–674 (2005). 10.1038/nature03395 15800630

[41] Lee J. Y. Investigating the sequence-dependent mechanical properties of DNA nicks for applications in twisted DNA nanostructure design. Nucleic Acids Res 47, 93–102 (2019). 10.1093/nar/gky1189 30476210 PMC6326809

[42] Reymer A., Zakrzewska K. & Lavery R. Sequence-dependent response of DNA to torsional stress: a potential biological regulation mechanism. Nucleic Acids Res 46, 1684–1694 (2018). 10.1093/nar/gkx1270 29267977 PMC5829783

[43] Yang J. Structures of CTCF-DNA complexes including all 11 zinc fingers. Nucleic Acids Res 51, 8447–8462 (2023). 10.1093/nar/gkad594 37439339 PMC10484683

[44] Hutchison C. A. 3rd Mutagenesis at a specific position in a DNA sequence. J Biol Chem 253, 6551–6560 (1978). 681366

[45] Botstein D. & Shortle D. Strategies and applications of in vitro mutagenesis. Science 229, 1193–1201 (1985). 10.1126/science.2994214 2994214

[46] Hill D. E., Hope I. A., Macke J. P. & Struhl K. Saturation mutagenesis of the yeast his3 regulatory site: requirements for transcriptional induction and for binding by GCN4 activator protein. Science 234, 451–457 (1986). 10.1126/science.3532321 3532321

[47] Takeda Y., Sarai A. & Rivera V. M. Analysis of the sequence-specific interactions between Cro repressor and operator DNA by systematic base substitution experiments. Proc Natl Acad Sci U S A 86, 439–443 (1989). 10.1073/pnas.86.2.439 2911590 PMC286485

[48] Sarai A. & Takeda Y. Lambda repressor recognizes the approximately 2-fold symmetric half-operator sequences asymmetrically. Proc Natl Acad Sci U S A 86, 6513–6517 (1989). 10.1073/pnas.86.17.6513 2771938 PMC297874

[49] Carrasco Pro S. Widespread perturbation of ETS factor binding sites in cancer. Nat Commun 14, 913 (2023). 10.1038/s41467-023-36535-8 36808133 PMC9938127

[50] Graves B. J., Gillespie M. E. & McIntosh L. P. DNA binding by the ETS domain. Nature 384, 322 (1996). 10.1038/384322a0 8934514

[51] Donaldson L. W., Petersen J. M., Graves B. J. & McIntosh L. P. Solution structure of the ETS domain from murine Ets-1: a winged helix-turn-helix DNA binding motif. EMBO J 15, 125–134 (1996). 8598195 PMC449924

[52] Garvie C. W., Hagman J. & Wolberger C. Structural studies of Ets-1/Pax5 complex formation on DNA. Mol Cell 8, 1267–1276 (2001). 10.1016/s1097-2765(01)00410-5 11779502

[53] Jiang B., Liu J. S. & Bulyk M. L. Bayesian hierarchical model of protein-binding microarray k-mer data reduces noise and identifies transcription factor subclasses and preferred k-mers. Bioinformatics 29, 1390–1398 (2013). 10.1093/bioinformatics/btt152 23559638 PMC3661050

[54] Kim J. L., Nikolov D. B. & Burley S. K. Co-crystal structure of TBP recognizing the minor groove of a TATA element. Nature 365, 520–527 (1993). 10.1038/365520a0 8413605

[55] Patikoglou G. A. TATA element recognition by the TATA box-binding protein has been conserved throughout evolution. Genes Dev 13, 3217–3230 (1999). 10.1101/gad.13.24.3217 10617571 PMC317201

[56] Xu Y., McSally J., Andricioaei I. & Al-Hashimi H. M. Modulation of Hoogsteen dynamics on DNA recognition. Nat Commun 9, 1473 (2018). 10.1038/s41467-018-03516-1 29662229 PMC5902632

[57] Nikolova E. N. Transient Hoogsteen base pairs in canonical duplex DNA. Nature 470, 498–502 (2011). 10.1038/nature09775 21270796 PMC3074620

[58] Shi H. Revealing A-T and G-C Hoogsteen base pairs in stressed protein-bound duplex DNA. Nucleic Acids Res 49, 12540–12555 (2021). 10.1093/nar/gkab936 34792150 PMC8643651

[59] Remenyi A. Crystal structure of a POU/HMG/DNA ternary complex suggests differential assembly of Oct4 and Sox2 on two enhancers. Genes Dev 17, 2048–2059 (2003). 10.1101/gad.269303 12923055 PMC196258

[60] Malaga Gadea F. C. & Nikolova E. N. Structural Plasticity of Pioneer Factor Sox2 and DNA Bendability Modulate Nucleosome Engagement and Sox2-Oct4 Synergism. J Mol Biol 435, 167916 (2023). 10.1016/j.jmb.2022.167916 36495920 PMC10184184

[61] O'Dwyer M. R. Nucleosome fibre topology guides transcription factor binding to enhancers. Nature 638, 251–260 (2025). 10.1038/s41586-024-08333-9 39695228 PMC11798873

[62] Soufi A. Pioneer transcription factors target partial DNA motifs on nucleosomes to initiate reprogramming. Cell 161, 555–568 (2015). 10.1016/j.cell.2015.03.017 25892221 PMC4409934

[63] Guo J. & Zhou H. X. Protein Allostery and Conformational Dynamics. Chem Rev 116, 6503–6515 (2016). 10.1021/acs.chemrev.5b00590 26876046 PMC5011433

[64] Puc J., Aggarwal A. K. & Rosenfeld M. G. Physiological functions of programmed DNA breaks in signal-induced transcription. Nat Rev Mol Cell Biol 18, 471–476 (2017). 10.1038/nrm.2017.43 PMC5854152 28537575

[65] Zhu W. DNA mutagenesis driven by transcription factor competition with mismatch repair. Cell 188, 5735–5747 e5715 (2025). 10.1016/j.cell.2025.07.003 40738104 PMC12327807

[66] Sabarinathan R., Mularoni L., Deu-Pons J., Gonzalez-Perez A. & Lopez-Bigas N. Nucleotide excision repair is impaired by binding of transcription factors to DNA. Nature 532, 264–267 (2016). 10.1038/nature17661 27075101

[67] Wu W. Neuronal enhancers are hotspots for DNA single-strand break repair. Nature 593, 440444 (2021). 10.1038/s41586-021-03468-5 PMC9827709 33767446

[68] Iwakura Y. Elevation of EGR1/zif268, a Neural Activity Marker, in the Auditory Cortex of Patients with Schizophrenia and its Animal Model. Neurochem Res 47, 2715–2727 (2022). 10.1007/s11064-022-03599-9 35469366

[69] Sams D. S. Neuronal CTCF Is Necessary for Basal and Experience-Dependent Gene Regulation, Memory Formation, and Genomic Structure of BDNF and Arc. Cell Rep 17, 2418–2430 (2016). 10.1016/j.celrep.2016.11.004 27880914

[70] Cao J. Harnessing a previously unidentified capability of bacterial allosteric transcription factors for sensing diverse small molecules in vitro. Sci Adv 4, eaau4602 (2018). 10.1126/sciadv.aau4602 30498782 PMC6261655

[71] Mao P. ETS transcription factors induce a unique UV damage signature that drives recurrent mutagenesis in melanoma. Nat Commun 9, 2626 (2018). 10.1038/s41467-018-05064-0 29980679 PMC6035183

[72] Frigola J., Sabarinathan R., Gonzalez-Perez A. & Lopez-Bigas N. Variable interplay of UV-induced DNA damage and repair at transcription factor binding sites. Nucleic Acids Res 49, 891–901 (2021). 10.1093/nar/gkaa1219 33347579 PMC7826277

[73] Sivapragasam S. Molecular basis of UV lesion binding and repair inhibition by ETS-family transcription factors. bioRxiv (2026). 10.64898/2026.01.13.699325 PMC13401050 42500821

[74] Legrand A. J., Choul-Li S., Villeret V. & Aumercier M. Poly(ADP-ribose) Polyremase-1 (PARP-1) Inhibition: A Promising Therapeutic Strategy for ETS-Expressing Tumours. Int J Mol Sci 24 (2023). 10.3390/ijms241713454 PMC10487777 37686260

[75] Oberbeckmann E., Quililan K., Cramer P. & Oudelaar A. M. In vitro reconstitution of chromatin domains shows a role for nucleosome positioning in 3D genome organization. Nat Genet 56, 483492 (2024). 10.1038/s41588-023-01649-8 PMC10937381 38291333

[76] Tran K. D. & Dutta A. In Vitro Assembly of Nucleosomes for Binding/Remodeling Assays. Methods Mol Biol 2919, 1–18 (2025). 10.1007/978-1-0716-4486-7_1 40257554

[77] Duan M. High-resolution mapping demonstrates inhibition of DNA excision repair by transcription factors. Elife 11 (2022). 10.7554/eLife.73943 PMC8970589 35289750

[78] Kaiser V. B. Mutational bias in spermatogonia impacts the anatomy of regulatory sites in the human genome. Genome Res 31, 1994–2007 (2021). 10.1101/gr.275407.121 34417209 PMC8559717

[79] Reijns M. A. M. Lagging-strand replication shapes the mutational landscape of the genome. Nature 518, 502–506 (2015). 10.1038/nature14183 25624100 PMC4374164

[80] Nithun R. V. Deciphering the Role of the Ser-Phosphorylation Pattern on the DNA-Binding Activity of Max Transcription Factor Using Chemical Protein Synthesis. Angew Chem Int Ed Engl 62, e202310913 (2023). 10.1002/anie.202310913 37642402

[81] Nithun R. V. Site-Specific Acetylation of the Transcription Factor Protein Max Modulates Its DNA Binding Activity. ACS Cent Sci 10, 1295–1303 (2024). 10.1021/acscentsci.4c00686 38947213 PMC11212134

[82] Badis G. Diversity and complexity in DNA recognition by transcription factors. Science 324, 1720–1723 (2009). 10.1126/science.1162327 19443739 PMC2905877

[83] Berger M. F. & Bulyk M. L. Universal protein-binding microarrays for the comprehensive characterization of the DNA-binding specificities of transcription factors. Nat Protoc 4, 393–411 (2009). 10.1038/nprot.2008.195 19265799 PMC2908410

[84] Berger M. F. Compact, universal DNA microarrays to comprehensively determine transcription-factor binding site specificities. Nat Biotechnol 24, 1429–1435 (2006). 10.1038/nbt1246 16998473 PMC4419707

[85] Kabsch W. Xds. Acta Crystallogr D Biol Crystallogr 66, 125–132 (2010). 10.1107/S0907444909047337 20124692 PMC2815665

[86] Chen V. B. MolProbity: all-atom structure validation for macromolecular crystallography. Acta Crystallogr D Biol Crystallogr 66, 12–21 (2010). 10.1107/S0907444909042073 20057044 PMC2803126

[87] Emsley P., Lohkamp B., Scott W. G. & Cowtan K. Features and development of Coot. Acta Crystallogr D Biol Crystallogr 66, 486–501 (2010). 10.1107/S0907444910007493 20383002 PMC2852313

[88] Adams P. D. PHENIX: a comprehensive Python-based system for macromolecular structure solution. Acta Crystallogr D Biol Crystallogr 66, 213–221 (2010). 10.1107/S0907444909052925 20124702 PMC2815670

[89] Tian C. ff19SB: Amino-Acid-Specific Protein Backbone Parameters Trained against Quantum Mechanics Energy Surfaces in Solution. J Chem Theory Comput 16, 528–552 (2020). 10.1021/acs.jctc.9b00591 31714766 PMC13071887

[90] Galindo-Murillo R. Assessing the Current State of Amber Force Field Modifications for DNA. J Chem Theory Comput 12, 4114–4127 (2016). 10.1021/acs.jctc.6b00186 27300587 PMC4980684

[91] Lee J. CHARMM-GUI supports the Amber force fields. J Chem Phys 153, 035103 (2020). 10.1063/5.0012280 32716185

[92] Amemiya H. M., Kundaje A. & Boyle A. P. The ENCODE Blacklist: Identification of Problematic Regions of the Genome. Sci Rep 9, 9354 (2019). 10.1038/s41598-019-45839-z 31249361 PMC6597582

[93] Ovek Baydar D. JASPAR 2026: expansion of transcription factor binding profiles and integration of deep learning models. Nucleic Acids Res 54, D184–D193 (2026). 10.1093/nar/gkaf1209 41325984 PMC12807658

